# Prevalence and risk factors for SARS-CoV-2 infection and seroprevalence among clinical and non-clinical staff in a national healthcare system

**DOI:** 10.1371/journal.pone.0257845

**Published:** 2021-09-30

**Authors:** Moza Alishaq, Andrew Jeremijenko, Zeina Al-Kanaani, Hanaa Nafady-Hego, Diana H. Jboor, Rosaline Saba, Jameela Al-Ajmi, Nasser Asad Alansari, Anil George Thomas, Sameera Bihi Fareh, Suni Vinoy, Maryam Nooh, Nadya Alanzi, Abdul-Badi Abou-Samra, Adeel Ajwad Butt

**Affiliations:** 1 Hamad Medical Corporation, Doha, Qatar; 2 Microbiology and Immunology Department, Faculty of Medicine, Assiut University, Assiut, Egypt; 3 Weill Cornell Medicine, Doha Qatar and New York NY, United States of America; Post Graduate Institute of Medical Education and Research, INDIA

## Abstract

**Background:**

While many studies have reported the rate and risk of SARS-CoV-2 infection among healthcare workers (HCWs), there are scant data regarding the impact of employment type and job grades upon such risk.

**Methods:**

We determined the rate of SARS-CoV-2 infection based on a positive nasopharyngeal swab (NPS) PCR among employees of a large national healthcare system. Antibody testing was performed on those who agreed to provide a blood sample. Using logistic regression analysis, we determined the risk of infection (PCR+) associated with demographic characteristics, job family and job grade.

**Results:**

We identified 35,075 staff (30,849 full-time, 4,226 outsourced) between March 1-October 31, 2020. Among full-time employees, 78.0% had a NPS (11.8% positive). Among outsourced staff, 94.4% had a NPS (31.1% positive). Antibody testing was performed on 33.9% full-time employees (13.0% reactive), and on 39.1% of the outsourced staff (47.0% reactive). PCR-positivity was higher among outsourced staff (31.0% vs. 18.3% in non-clinical and 9.0% in clinical full-time employees) and those in the low-grade vs. mid-grade and high-grade job categories. Male sex (OR 1.88), non-clinical job family (OR 1.21), low-grade job category (OR 3.71) and being an outsourced staff (OR 2.09) were associated with a higher risk of infection.

**Conclusion:**

HCWs are a diverse population with varying risk of infection. Clinical staff are at a lower risk likely due to increased awareness and infection prevention measures. Risk is higher for those in the lower socioeconomic strata. Infection is more likely to occur in non-healthcare setting than within the healthcare facilities.

## Introduction

Persons working in healthcare facilities are at a higher risk of SARS-CoV-2 infection. This is due to their potential exposure in the community plus added exposure to symptomatic and critically ill patients in acute and intensive care settings in the healthcare facilities. Persons working in healthcare facilities are a heterogenous group, which include frontline clinical care providers, allied health professionals, and clinical and non-clinical support staff. These groups may also have variable level of exposure in the community due to social and economic circumstances. Accordingly, their overall exposure and risk of infection may vary significantly which may lead to variable rates of symptomatic infection and seropositivity [1]. Indeed, the reported seroprevalence among healthcare workers is highly variable and ranges from a low of 1.6% to a high of 17%, with higher exposure risk associated with higher seroprevalence rates [1–4]. Most published studies are limited by small sample size, geographically limited study population or convenience testing of workers. Job category within the healthcare workforce and job grade, which can serve as a surrogate of socioeconomic status, have rarely been studied in the context of SARS-CoV-2 infection rates and risk. Among healthcare workers, seroprevalence varies between 10–24%, with higher rates of infection noted among some non-clinical staff (e.g. cleaners) and lower rates noted among physicians [5–8]. A large proportion of infection among healthcare workers are asymptomatic and diagnosed through routine serologic testing or as part of research studies [6].

Qatar is a modern nation-state with unique population and workforce demographics which are quite different from most other countries. Among its 2.8 million residents, approximately 85% are expatriate workers. Due to this, the overall population is skewed heavily towards a younger male population, a sizeable proportion of whom work as craft and manual workers [9, 10]. Qatar has high SARS-CoV-2 infection rates, but one of the lowest case fatality rates in the world due to an aggressive testing, contact tracing, isolation and early treatment policies [11, 12]. In a large study of ten communities in Qatar, the pooled seropositivity rate was 66%, with severe or critical infection occurring in only 0.2% of the infected persons among craft and manual workers, while the seroprevalence in the urban communities was 13.3% [13, 14]. Among healthcare workers in Qatar, we previously reported that 10.6% had tested positive for SARS-CoV-2 by a nasopharyngeal swab RT-PCR [15]. The seroprevalence of SARS-CoV-2 infection among all employees of the healthcare system is unknown. We sought to determine the prevalence and risk factors for infection among full time staff and contracted employees through outsourced services working at Qatar’s largest public healthcare system.

## Methods

### Setting and participants

The study was conducted at Hamad Medical Corporation (HMC), Qatar, the largest integrated public health provider in the State of Qatar. HMC provides approximately 85% of inpatient bed capacity in the State of Qatar and operates 14 healthcare facilities that include secondary and tertiary care general hospitals and specialty care hospitals. All of HMC’s physicians, nurses, allied health professionals and administrative staff are full-time employees, while certain maintenance functions (laundry, catering, housekeeping) are outsourced and performed by employees of contracted companies. For the purpose of this study, we classified the job families of our study population into clinical care staff (physicians, nurses and allied health professionals) and non-clinical staff (administrative staff, non-clinical executive leadership and other support services staff) and contracted staff. Job grades were categorized into high-grade, mid-grade and low grade based on the average educational and professional qualifications and expected remuneration. Employees who were tested multiple times and those with more than one positive test were counted only once.

### Testing

The study period was March 1, 2020 through October 31^st^, 2020. All full-time employees and outsourced staff were offered PCR testing on a nasopharyngeal swab. Testing was mandatory for persons with symptoms compatible with SARS-CoV-2 infection (symptoms suggestive of upper or lower respiratory tract infection) and contacts of those with confirmed infection. Serum antibody testing for SARS-CoV-2 antibodies was performed on those who requested it or were referred by their clinical care provider. All laboratory testing was conducted at Hamad Medical Corporation central laboratory following standardized protocols. The laboratory is accredited by the College of American Pathologists.

PCR testing was performed using real-time reverse-transcription PCR [RT-qPCR] using the TaqPath™ COVID-19 Combo Kit [Thermo Fisher Scientific, USA], AccuPower SARS-CoV-2 Real-Time RT-PCR Kit [Bioneer, Korea] or Roche cobas® SARS-CoV-2 Test [Roche, Switzerland]. Testing for SARS-CoV-2-specific antibodies in the serological samples was performed using an electrochemiluminescence immunoassay, the Roche Elecsys^®^ Anti-SARS-CoV-2 [Roche, Switzerland]. Results’ interpretation was per manufacturer’s instructions: reactive for cutoff index ≥1.0 and non-reactive for cutoff index <1.0.

### Data collection

Demographic and clinical data were collected from the electronic medical records. Job grade (indicative of education status, training, experience and a surrogate marker of remuneration) and job family were obtained from the Human Resources Department. Job grades are listed as numerical values, with higher numerical values indicative of seniority. While financial remuneration is determined by numerous factors, higher grades generally indicate higher overall remuneration.

### Analyses

We determined the number and proportion of full time employees and outsourced staff who tested positive for SARS-CoV-2 PCR on a nasopharyngeal swab. We also determined the seroprevalence among those who provided a blood sample for testing. We used logistic regression analysis to determine the odds ratio and 95% confidence intervals for demographic and work related characteristics predictive of PCR-positive infection.

### Ethical approval

The study was approved by the Institutional review Board at Hamad Medical Corporation. The study was granted a waiver of informed consent requirement.

## Results

**Fig 1:**
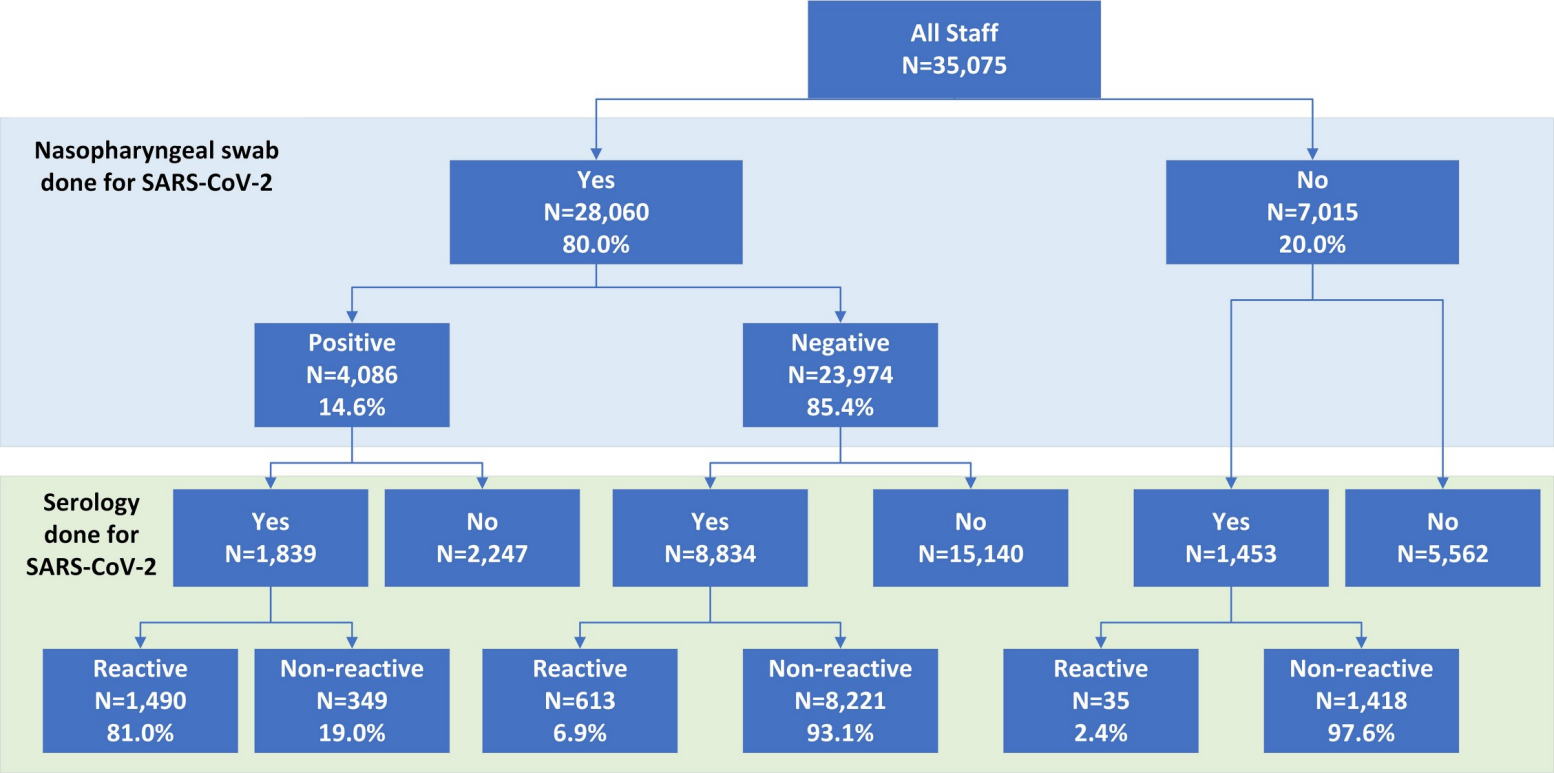
A flowsheet of all staff (full-time and contracted) who were tested for SARS-CoV-2 infection.

We identified a total of 35,075 staff (30,849 full-time employees and 4,226 outsourced staff) during the study period. Among all staff, 28,060 (80.0%) had a nasopharyngeal swab performed for SARS-CoV-2 PCR testing with 4,086 (14.6% of those tested) being positive. Among the PCR-positive group, 1,839 (45.0%) were tested for the presence of SARS-CoV-2 antibodies and 1,490 (81% of those tested) were reactive. Among the 23,974 staff with a negative nasopharyngeal swab by PCR, 8,834 (36.8%) were tested for antibodies and 613 (6.9%) were reactive. Among the 7,015 persons without a nasopharyngeal swab, 1,453 (20.7%) provided a blood sample for antibody testing and 35 (2.4% of those tested) were reactive **(Fig 1)**.

**Fig 2:**
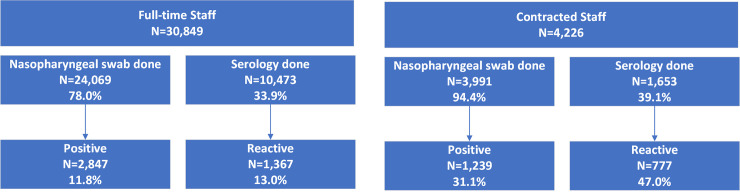
A flowsheet of full-time and contracted who were tested for SARS-CoV-2 infection by PCR on a nasopharyngeal swab and for SARS-CoV-2 antibody.

Among 30,849 full-time employees, 24,069 (78.0%) had a nasopharyngeal swab performed with 2,847 (11.8% of those tested) testing positive. Among 4,226 outsourced staff, 3,991 (94.4%) had a nasopharyngeal swab performed with 1,239 (31.1%) testing positive. Antibody testing was performed on 10,473 (33.9%) full-time employees with 1,367 of those tested (13.0%) being reactive, and on 1,653 (39.1%) of the outsourced staff of which 777 (47.0%) were reactive **(Fig 2)**.

Baseline characteristics of staff who provided a nasopharyngeal swab for PCR and who tested positive are provided in **Table 1**. Positivity by PCR was higher among males compared to females (19.5% vs. 9.5%) and highest among those <30 years old (20.0%) compared with older age groups. Outsourced staff had the highest PCR positivity (31.0%) compared with 18.3% among the non-clinical full time employees and 9.0% among the clinical full time employees. Similarly, highest PCR positivity was observed among those in the low grade job category compared with mid-grade and high-grade job categories.

**Table 1:** PCR positivity and seropositivity rates.

	Total	PCR Swabbed	PCR positivity^1^	Tested for antibody	Seropositivity^1^(%)
	(N = 35,075)	(n = 28,060)	(%)	(n = 12,126)	
Sex					
Male	17,672	14,210	19.49%	5,511	25.82%
Female	17,282	13,823	9.52%	6,597	10.81%
Missing	121	27	3.70%	18	11.11%
Age groups					
<30 years	5,385	4,647	20.01%	1,780	27.98%
30–59 years	28,730	22,769	13.53%	10,006	16.03%
> 60 years	947	644	11.80%	340	13.82%
Missing	13	0	-		
Job family					
Clinical	20,840	16,846	9.03%	7,736	10.03%
Non-Clinical	9,913	7,208	18.35%	2,730	21.61%
Outsourced^2^	4,226	3,991	31.04%	1,653	46.64%
Job Grade^2^					
High-grade	4,002	2,765	6.98%	1,438	7.23%
Mid-grade	21,923	17,489	10.13%	7,644	11.05%
Low-grade	4,830	3,800	23.12%	1,384	30.13%
Missing	94	15	13.33%	7	14.29%

^1^ Among those tested.

^2^ The 4,226 outsourced staff is categorized under “Job family”.

In multivariable logistic regression analysis, older age groups were associated with a lower risk of infection compared with the younger age group (<30 years old) **(Table 2)**. Male sex (OR 1.88; 95% CI 1.74,2.02), non-clinical job family (OR 1.21; 95% CI 1.09,1.34), low grade job category (OR 3.71; 95% CI 3.11,4.11) and being an outsourced staff (OR 2.09; 95% CI 1.88,2.33) were associated with a higher risk of infection **(Table 2)**. Since age can be an indirect and surrogate marker of seniority, higher grade, and accommodation status, we recalculated the odds ratios after excluding age as a covariate. The results for the remaining covariates were essentially unchanged **(S1 Table)**.

**Table 2:** 

Characteristic	Unadjusted Odds Ratio	95% CI	P Value	Adjusted Odds Ratio	95% CI	P Value
Age			0.007			0.6
Less than 30	Ref.	Ref.		Ref.	Ref.	
30–59	0.57	0.53–0.62		1.06	0.96–1.17	
60+	0.42	0.33–0.53		0.93	0.71–1.21	
Gender			0.02			<0.001
Female	Ref.	Ref.		Ref.	Ref.	
Male	2.25	2.10–2.41		1.88	1.74–2.02	
Job Family*			0.04			0.001
Clinical	Ref.	Ref.		Ref.	Ref.	
Non-Clinical	2.81	2.62–3.00		1.21	1.09–1.34	
Job Grades*			0.06			<0.001
High grades	Ref.	Ref.		Ref.	Ref.	
Mid grades	1.73	1.48–2.02		2.05	1.76–2.40	
Lower grades	6.03	5.17–7.03		3.71	3.11–4.11	
Employer			0.04			<0.001
HMC	Ref.	Ref.		Ref.	Ref.	
Outsourced	4.08	3.77–4.40		2.09	1.88–2.33	

## Discussion

We provide a detailed analysis of the prevalence and risk factors for SARS-CoV-2 infection in a national healthcare system. We found older age, female sex, clinical job family and higher job grades to be associated with a lower prevalence and a lower risk of infection.

Healthcare workers are presumed to be at a higher risk of infection in a pandemic setting. This belief stems from their dual exposure in the community and in the hospitals. However, we and others have shown a lower incidence and risk of SARS-CoV-2 infection among HCWs [1, 2, 15]. This is most likely due to the heightened awareness and strict infection prevention measures adopted by HCWs in response to the pandemic. However, HCWs are a heterogenous population and the risk of infection may vary based on multiple factors. Perhaps the most important of these is the level of community exposure, which in turn depends on the social, cultural, and economic factors and personal attitudes towards infection prevention measures [16]. Our finding of a higher rate and risk of infection among employees with lower job grades is likely a reflection of personal accommodation and exposure. Those with lower income are more likely to live in shared accommodations and thus at a higher risk of exposure. On average, non-clinical workers and outsourced staff tend to be in the lower income strata and are more likely to live in crowded accommodations, thus increasing the risk of exposure and infection.

We found men to be at a significantly higher risk of infection. This is likely due to the increased mobility and social interactions among men in many cultures, particularly in the Middle East. Whether there are difference in attitudes towards wearing a mask or practicing physical distancing measures is unknown. Such differences could be additional reasons for the different rate and risk of infection based on sex.

The risk of infection among the outsources staff was twice as much as compared with full time employees. The contracted staff are employed predominantly in the unskilled professions and are therefore in the lower socioeconomic strata. Most contracted staff live in shared accommodations and are thus at a high risk of exposure and infection. Our previous work provides credence to this inference since the rate and risk of infection among HCWs was mostly due to non-work related exposure [15, 16].

The strengths of our study include a large study population spanning an entire country. Testing was performed free of cost and an overwhelming proportion of HCWs provided samples for testing. Limitations include lack of information on exposure to confirmed cases and individual living conditions.

In conclusion, HCWs are a diverse population with varying risk of exposure and infection. Clinical care providers appear to be at a lower risk likely due to increased awareness and precautions taken to avoid infection. Risk is higher for those in the lower socioeconomic strata. Infection is more likely to occur in non-healthcare setting than within the healthcare facilities.

## Supporting information

S1 Table Risk factors for SARS-CoV-2 infection among healthcare staff after excluding age as a covariate. (DOCX)
