# Supplementary material for: Prevalence and risk factors for SARS-CoV-2 infection and seroprevalence among clinical and non-clinical staff in a national healthcare system
Source: PLoS One. 2021 Sep 30;16(9):e0257845. doi: 10.1371/journal.pone.0257845 (PMC8483404; doi:10.1371/journal.pone.0257845)
Supplement: S1 Table — (DOCX) [file pone.0257845.s001.docx]

S1 Table. Risk factors for SARS-CoV-2 infection among healthcare staff after excluding age as a covariate.

| Characteristic | Adjusted Odds Ratio | 95% CI | P Value* |
| --- | --- | --- | --- |
| Gender  Female  Male | Ref.  1.88 | Ref.  1.74-2.02 | <0.001 |
| Job Family  Clinical  Non-Clinical | Ref.  1.20 | Ref.  1.09-1.33 | <0.001 |
| Job Grades  High grades  Mid grades  Lower grades | Ref.  2.06  3.73 | Ref.  1.77-2.41  3.12-4.46 | <0.001 |
| Employer  HMC  Outsource | Ref.  2.04 | Ref.  1.85-2.26 | <0.001 |
